# Evaluation of the impact of collaborative work by teams from the National Medical Residency Committee and the Brazilian Society of Neurosurgery. Retrospective and prospective study: Avaliação do impacto do trabalho conjunto das equipes da Comissão Nacional de Residência Médica e da Sociedade Brasileira de Neurocirurgia. Estudo retrospectivo e prospectivo

**DOI:** 10.1590/1516-3180.2015.9603001

**Published:** 2015-10-09

**Authors:** Renato Antunes dos Santos, Linda Snell, Maria do Patrocínio Tenório Nunes

**Affiliations:** I MD. Doctoral student, Universidade de São Paulo (USP), São Paulo, Brazil. Psychiatrist at the University Hospital of Brasília, Universidade de Brasília (UnB), Brasília, Brazil. Visiting Professor at McGill’s Centre for Medical Education, Montreal-Canada.; II MD. Professor of Medicine, Core Member of McGill’s Centre for Medical Education and Senior Clinician Educator at the Royal College of Physicians and Surgeons of Canada.; III MD, PhD. Full and Associate Professor, Discipline of General Practice and Propaedeutics, Department of Internal Medicine, Universidade de São Paulo (USP), São Paulo, Brazil.

**Keywords:** Education, medical, Internship and residency, Educational measurement, Program evaluation, Neurosurgery

## Abstract

**CONTEXT AND OBJECTIVE:**

Training for specialist physicians in Brazil can take place in different ways. Closer liaison between institutions providing this training and assessment and health care services may improve qualifications. This article analyzes the impact of closer links and joint work by teams from the National Medical Residency Committee (Comissão Nacional de Residência Médica, CNRM) and the Brazilian Society of Neurosurgery (Sociedade Brasileira de Neurocirurgia, SBN) towards evaluating these programs.

**DESIGN AND SETTING:**

Retrospective and prospective study, conducted in a public university on a pilot project developed between CNRM and SBN for joint assessment of training programs across Brazil.

**METHODS:**

The literature in the most relevant databases was reviewed. Documents and legislation produced by official government bodies were evaluated. Training locations were visited. Reports produced about residency programs were analyzed.

**RESULTS:**

Only 26% of the programs were immediately approved. The joint assessments found problems relating to teaching and to functioning of clinical service in 35% of the programs. The distribution of programs in this country has a strong relationship with the Human Development Index (HDI) of the regions and is very similar to the distribution of specialists.

**CONCLUSION:**

Closer collaboration between the SBN and CNRM had a positive impact on assessment of neurosurgery medical residency across the country. The low rates of direct approval have produced modifications and improvements to the quality of teaching and care (services). Closer links between the CNRM and other medical specialties have the capability to positively change the structure and function of specialty training in Brazil.

## RESUMO

**CONTEXTO E OBJETIVO:**

A formação do médico especialista no Brasil pode ocorrer por diferentes vias. A aproximação das instituições que realizam essas formações e avaliam os médicos e as instituições de saúde pode trazer benefícios na qualificação. Este artigo analisa o impacto dessa aproximação e o trabalho conjunto das equipes da Comissão Nacional de Residência Médica (CNRM) e da Sociedade Brasileira de Neurocirurgia (SBN) na avaliação desses programas.

**TIPO DE ESTUDO E LOCAL:**

Estudo retrospectivo e prospectivo, conduzido em uma universidade pública, sobre projeto piloto elaborado entre CNRM e SBN na avaliação conjunta dos programas de treinamento pelo Brasil.

**MÉTODOS:**

Revisão de literatura nas principais bases de dados, documentos e legislações produzidas por órgãos oficiais governamentais, visitas aos locais de formação e análise dos relatórios e pareces produzidos sobre os programas de residência médica.

**RESULTADOS:**

Apenas 26% dos programas foram aprovados diretamente. As avaliações conjuntas encontraram problemas relacionados ao ensino e ao funcionamento do serviço em cerca de 35% dos programas. A distribuição dos programas no país tem forte relação com o Índice de Desenvolvimento Humano (IDH) das regiões e é muito semelhante à distribuição dos especialistas.

**CONCLUSÃO:**

A aproximação da SBN com a CNRM teve impacto positivo na avaliação das residências médicas em neurocirurgia no país. Os índices baixos de aprovação direta forçaram a realização de modificações e melhorias na qualidade de ensino e assistência (serviço). A aproximação da CNRM e das demais especialidades médicas pode alterar positivamente a estrutura e o funcionamento da formação médica no país.

## INTRODUCTION

The world is currently facing a lack and poor distribution of healthcare professionals.^1^ Many institutions and official bodies around the world have been studying and planning workforce supply and strategies, such as the European Union’s Joint Action on Health Workforce Planning and Forecasting,^2^ Australian Medical Advisory Committee,^3^ Netherlands Advisory Committee on Medical Manpower Planning, Belgian Health Workforce Planning Unit, International Medical Workforce Collaborative and others. The task of determining the distribution, specialist types, quantity and quality of healthcare professionals has been started around the world, in order to plan the future healthcare workforce.^4^^,^^5^^,^^6^

The training process for healthcare professionals is very long and complex. For physicians, the time span from the beginning of medical school until entering the labor market may be more than 12 years.^7^ Understanding the specialization processes and distribution of medical specialists seems to be essential for good workforce planning.

Aside from all the general complexity, there are different mechanisms for training medical specialists in Brazil. There are also singular regulations for the accreditation process of medical specialization:^8^^,^^9^

1. Medical Residency. This is considered to be the gold standard method with nationally unified laws, rules and criteria. Medical residency is administered by the National Medical Residency Committee (Comissão Nacional de Residência Médica, CNRM), which is located within the Ministry of Education and is composed of representatives of the Ministry of Education, Ministry of Health, Brazilian Medical Association, Medical Union, Federal Medical Council, National Residents Association, Municipal Health Departments and State Health Departments, thus constituting the plenary body of the CNRM.
2. Medical Specialization Courses. These are courses accredited by specialist medical societies that are used to train new specialists. The models for such courses have variable criteria that are approved by the Scientific Council of the Brazilian Medical Association. This training process historically has had a significant role in Brazil. It usually involves the same length of training and part of the content of medical residency programs. The specialist medical societies apply an evaluation process at the end of the training period, although with variable criteria.

After new graduates receive the formal degree of physician, Brazilian law allows them to practice any medical specialty, as long as they feel able to do so. After a validated and well-documented period of a few years of practice in well-reputed services, under the supervision of experts, physicians can apply to take tests administered by the Brazilian Medical Association, in order to receive a certificate in a specialty. The same certificate is validated at the end of medical residency training.

Regardless of the path taken, physicians’ certificates need to be registered at the Federal Medical Council, which, according to a specific law,^10^ has the power to regulate and supervise medical practice.^11^

Even today, the Brazilian healthcare and educational authorities are still trying to identify the real number of specialists in the country and the actual requirements in each of the 53 medical specialties recognized in this country,^12^ in accordance with epidemiological data and international parameters. It is also necessary to bring training methods together through recognizing historical Brazilian medical practices and specialist training processes.

The CNRM^13^^,^^14^ has started to work in this direction with the specialist medical associations. The intention was to unify the training process, so as to avoid the possibility that different learning material (knowledge, skills and attitudes) might be provided for the same specialist qualification. Among the 53 recognized Brazilian medical specialties, neurosurgery was chosen for the pilot project of this study.

Neurosurgery was chosen because the Brazilian Society of Neurosurgery (Sociedade Brasileira de Neurocirurgia, SBN) was willing to participate and because access to its Neurosurgery Assessment Committee was facilitated. The SBN has a well-organized evaluation process that covers institutions, their residency programs and residents. It was taken into account that it is virtually impossible to practice neurosurgery without formal training and official recognition.

The CNRM and SBN started by working together, planning an assessment tool and visiting neurosurgery residency programs and neurosurgery services throughout the country.

## OBJECTIVE

This study had the aim of assessing the current situation of medical residency programs in neurosurgery in Brazil, in the light of the partnership between the CNRM and SBN. It also aimed to analyze the locoregional distribution of medical residency programs within neurosurgery, the distribution of specialists in this field and the current situation now that the SBN-CNRM collaboration has come into practice.

## METHODS

This study began with a review of the available scientific literature, through searching regional databases (Lilacs, SciELO and Bireme) and global databases (PubMed and Web of Science) with regard to the external medical residency evaluation process implemented by government bodies, professional associations, scientific societies, etc. In addition, any available articles and legislation relating to evaluation, regulation and supervision that had been published by government bodies such as the Ministries of Health and Education, or by medical associations, the Federal Medical Council or other similar entities, were also included.

Focusing on the quality of neurosurgical residency, a single assessment instrument was developed by the CNRM and SBN in relation to the supervised in-service educational process, which aimed to investigate the following factors: infrastructure and characteristics of the institution; educational program; care profile; staff qualifications; whether the staff worked exclusively for the institution in question; clinical demands (number and variety of cases in accordance with the competencies to be developed over the period of the residency program); and development (apprenticeship) of medical residents.

The process of on-site educational evaluation took place as follows:

1. The instrument was sent to the institutions to be evaluated in accordance with criteria that had been established jointly by CNRM and SBN.
2. No more than two weeks after the instrument had been sent out, institutions across the country were visited by at least two appraisers (at least one person from CNRM and another from SBN), in accordance with a predetermined schedule. The evaluation team assessed the conditions of the wards, outpatient clinics, surgical center, radiological unit, hemodynamic unit, laboratory, emergency room, intensive care unit, all necessary tools (including microscopes), numbers and types of operations performed within the last six months, library provision, access to electronic libraries and compliance with theoretical programs and legislation.
3. The evaluation team held meetings separately with the management of each institution, the coordinators and supervisors of the medical residency programs and the medical residents for the purpose of ascertaining the strengths and weaknesses of the program.
4. A final report was produced by the evaluation team.
5. The reports thus produced were analyzed by a CNRM technical council, which deliberated on corrective measures to be proposed for residency programs.
6. The plenary body of the CNRM deliberated on the measures suggested by the technical council.
7. The institutions were notified of the measures that needed to be implemented over a certain period of time that was set by the plenary body of the CNRM.
8. Compliance with the changes was verified at the end of the period proposed by the plenary body of the CNRM.

We evaluated all the assessments that were made and all the opinions issued by the CNRM technical council, and attempted to check their impact on the recent history of each program and the consequences for the healthcare provided at the institution and for medical education decisions.

Meetings between representatives from SBN and CNRM were held to establish goals and work processes; to unify criteria and evaluation instruments; and to train the evaluation team. These meetings were held between April 2010 and February 2011.

The assessment visits took place between April 2011 and January 2014. Over this period, and until April 2014, the CNRM technical council analyzed the reports, the CNRM plenary body deliberated on the measures suggested and the institutions were notified of the actions to be implemented within the prescribed period. Finally, the changes implemented were checked at the end of the proposed period.

## RESULTS

Brazil has 26 states and one federal district. It was found that seven states do not have any neurosurgery programs: Acre, Amapá, Rondônia and Roraima (northern region); and Maranhão, Paraíba and Piauí (northeastern region). These seven states without neurosurgery programs correspond to regions with low Human Development Index (HDI).^15^

Neurosurgeons are distributed through the regions of Brazil: 94 in the north, 245 in the northeast, 171 in the center-west, 1197 in the southeast and 362 in the south, as demonstrated in previous studies.^16^^,^^17^ If these numbers are correlated with population sizes, the shortages of neurosurgeons can be better understood. The number of neurosurgeons per 100,000 inhabitants, according to Brazilian region, as defined by the Brazilian Institute for Geography and Statistics (Instituto Brasileiro de Geografia e Estatística, IBGE, 2010),^18^ is as follows: 0.59 in the north, 0.47 in the northeast, 1.49 in the southeast, 1.40 in the south and 1.22 in the center-west. The average number of neurosurgeons per 100,000 inhabitants for the whole country was 1.09.

Across the country, there were 154 vacancies for admission to medical residency programs in neurosurgery, distributed in 105 programs. Again dividing Brazil according to regions, the number of medical residency programs in neurosurgery per 100,000 inhabitants was 0.03 in the north, 0.03 in the northeast, 0.12 in the southeast, 0.10 in the south and 0.06 in the center-west. The average number of medical residency programs in neurosurgery per 100,000 inhabitants for the whole country was 0.08.^18^ Seventeen new neurosurgery programs were created during the study period and represented 14.6% of the total. Three were in the south, four in the southeast, three in the northeast, four in the center-west and three in the north.

**Figure 1.**
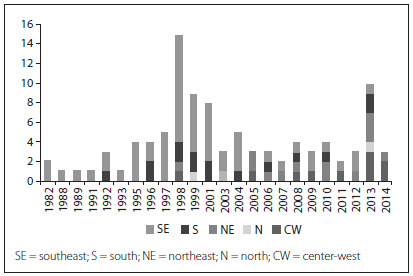
Neurosurgery residency training programs installed over the last 30 years in the five Brazilian regions.

Figure 1 represents the evolution of neurosurgery medical residency programs (NMRPs) installed in the five Brazilian regions over the last 30 years (i.e. since 1982). The shades of gray show that in the 1980s and 1990s, there was a heavy concentration of NMRPs in the southeastern and southern regions. Over that period, new program startups in other regions were exceptional. From 1999 to 2003, new NMRPs emerged in other regions. Over the past three years, the distribution of neurosurgery residency positions according to region has started to change. In particular, there was greater diversity in NMRP startups in 2012, with good responses in the northeastern and central-western regions.

The CNRM recognizes that 127 neurosurgery programs have existed historically. At the time of the present evaluation process, there were 105 programs. Twenty-three neurosurgery programs had been canceled before the data analysis process took place, or were canceled during it.

After the assessment, it was possible to approve 28 NMRPs without any restrictions, corresponding to 26.7% of the total. Twenty-two of these programs are located in the southeast, five in the south and one in the north. Table 1 shows the situation of the 105 neurosurgery programs after the CNRM/SBN evaluation. Thirty-seven programs were placed under supervision for correction of irregularities (Table 1): eight in the south, twenty in the southeast, five in the northeast, and two each in the center-west and north. Four neurosurgery programs had to be closed immediately due to lack of appropriate conditions for teaching and medical care: three in the southeast and one in the northeast. For one program that was placed under supervision by the technical board, corrections were made quickly and it was approved by the final CNRM plenary session. Four programs continued to be out of date by the end of the period covered by this study. Fourteen were still waiting for assessment visits and no impact or results can be presented because they were first visited just a few weeks before the project began and could not be evaluated within five years.

**Table 1.**
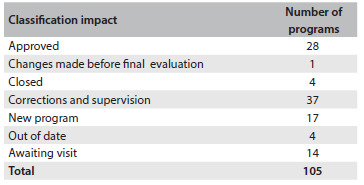
Situation of the 105 neurosurgery programs after evaluation by the National Medical Residency Committee (Comissão Nacional de Residência Médica, CNRM) and the Brazilian Society of Neurosurgery (Sociedade Brasileira de Neurocirurgia, SBN)

The most common problems found in the final reports of the evaluation, technical papers and CNRM plenary body are shown in Tables 2 and 3. These problems observed in the results can be divided into two groups:

- Service faults (structure, processes and outcomes), subdivided into the classical triad of healthcare service assessment drawn up by Donabedian,^18^^,^^19^ here with 19 different kinds of important problems found.
- Learning faults, concerning information and assessments used by the Ministry of Education to analyze medical residency, with 12 different important features found.

**Table 2.**
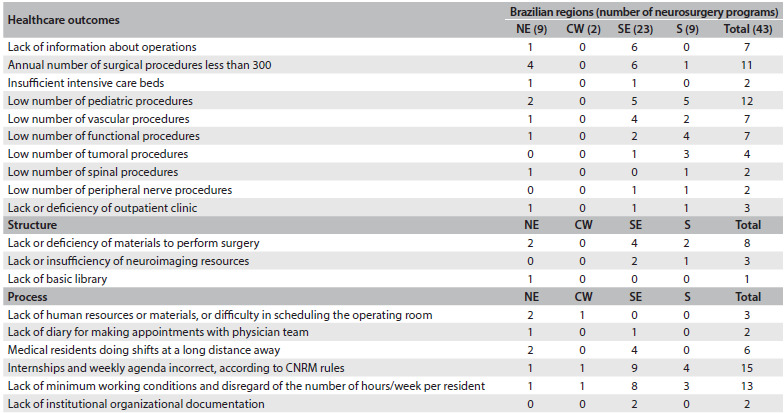
The most common problems found in the final reports from the evaluation, technical papers and National Medical Residency Committee (Comissão Nacional de Residência Médica, CNRM) plenary body regarding services

NE = northeast; CW = center-west; SE = southeast; S = south.

**Table 3.**
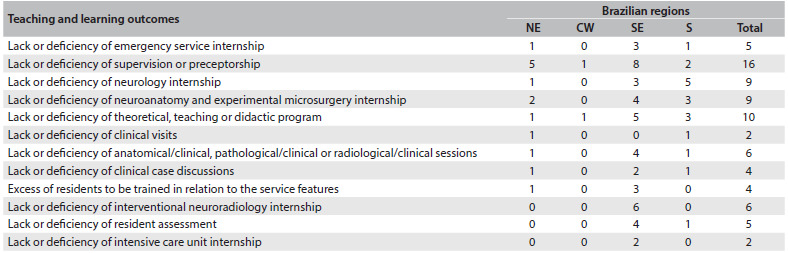
The most common problems found in the final reports from the evaluation, technical papers and National Medical Residency Committee (Comissão Nacional de Residência Médica, CNRM) plenary body regarding education

NE = northeast; CW = center-west; SE = southeast; S = south.

We defined structure as the materials (infrastructure, equipment and supplies) needed to conduct the residency program. Processes were defined as the relationship between human resource management, learning and healthcare. The results represented the ability and efficiency of surgery and clinical care.

The main problems found within teaching and learning related to deficiencies in internships, but there were also shortages of supervision and theoretical programs. Table 3 summarizes the teaching and learning problems identified during the CNRM/SBN evaluation.

## DISCUSSION

There is huge inequity with regard to economics, culture, health, educational performance and access, HDI and other factors among the the different regions.^15^^,^^20^ It is known that economics and HDI are linked to human capital, development and empowerment.^21^ There is a relationship between HDI and the numbers of neurosurgery programs and neurosurgeons. Neurosurgeons and neurosurgery residency vacancies are concentrated in the regions with best HDI. There seems to be a “snowball” of growth in inequity. Worse HDI correlates with services that have poor structure. This scenario is unattractive, and sometimes makes it impossible to practice neurosurgery specialization. The lack of specialists and structure make residency positions impossible, or even undesirable. Without medical residency training, there will be fewer specialists and less structure.^22^

There has been a federal government policy to induce residency programs since 2009, including for neurosurgery. This policy, called “ProResidência”, is an important initiative by the Ministries of Health and Education.^23^ ProResidência provides financial input from the Ministry of Health for residency. It is also responsible for putting into practice some discussion on planning and provision of doctors (generalists and specialists), and on the requirements of the population. The choice among the medical specialties for which residency programs might be induced is made according to the difficulty in hiring specific specialists in both the public and the private healthcare sectors.^24^

Other strategies are being implemented, such as supervision and assistance between institutions that want to improve their programs or start new ones and experienced universities.^25^ This strategy is a tentative government initiative, and its results have not yet been assessed. It is known as “matriciamento” (matrix support)^26^ and was designed to reformulate the process of healthcare work and used also for teaching and learning.

## CONCLUSION

Only about a quarter (26%) of the programs were immediately approved through this evaluation project. The evaluation team from the CNRM was well prepared to address the educational and legal aspects of medical residency in general, and this knowledge was brought to bear in these joint evaluations. The evaluations team of the SBN added value with regard to developing the content of the residency programs and technical issues relevant to the specialty.

CNRM and SBN have unified their evaluation criteria, showing very complex results, with large numbers of needs and weaknesses. It seems clear that although the isolated and parallel evaluation processes used in Brazil today are important, unification makes a difference with regard to improving the quality of teaching, clinical services and future medical practice.
